# Morphometric traits capture the climatically driven species turnover of 10 spruce taxa across China

**DOI:** 10.1002/ece3.1971

**Published:** 2016-01-25

**Authors:** He Li, GuoHong Wang, Yun Zhang, WeiKang Zhang

**Affiliations:** ^1^ State Key Laboratory of Vegetation and Environmental ChangeInstitute of BotanyChinese Academy of SciencesBeijing100093China; ^2^ University of the Chinese Academy of SciencesBeijing100049China

**Keywords:** Ecological amplitude, geographical distribution, interspecific and intraspecific variation, phylogeny, *Picea*

## Abstract

This study explored the relative roles of climate and phylogenetic background in driving morphometric trait variation in 10 spruce taxa in China. The study further addressed the hypothesis that these variations are consistent with species turnover on climatic gradients. Nine morphometric traits of leaves, seed cones, and seeds for the 10 studied spruce taxa were measured at 504 sites. These data were analyzed in combination with species DNA sequences from NCBI GenBank. We detected the effects of phylogeny and climate through trait‐variation‐based K statistics and phylogenetic eigenvector regression (PVR) analyses. Multivariate analyses were performed to detect trait variation along climatic gradients with species replacement. The estimated *K*‐values for the nine studied morphometric traits ranged from 0.19 to 0.68, and the studied environmental variables explained 39–83% of the total trait variation. Trait variation tended to be determined largely by a temperature gradient varying from wet‐cool climates to dry‐warm summers and, additionally, by a moisture gradient. As the climate became wetter and cooler, spruce species tended to be replaced by other spruces with smaller needle leaves and seeds but larger cones and seed scales. A regression analysis showed that spruce species tended to be successively replaced by other species, along the gradient, although the trends observed within species were not necessarily consistent with the overall trend. The climatically driven replacement of the spruces in question could be well indicated by the between‐species variation in morphometric traits that carry lower phylogenetic signal. Between‐species variation in these traits is driven primarily by climatic factors. These species demonstrate a narrower ecological amplitude in temperature but wider ranges on the moisture gradient.

## Introduction

It has been well documented that environmental preferences differ among species, leading to the frequent replacement of species or vegetation types along environmental gradients (Spalding 1890; MacArthur 1972). The observed climatic conditions under which a species can persist at the extreme limits of its distributional range mark the range of tolerance of the species. The range of tolerance (i.e., set of limiting environmental conditions) of a species is defined as the species' ecological amplitude (Ordonez et al. 2009). A species' ecological amplitude (also termed “niche breadth” in the older ecological literature) is, most likely, related to ecological strategies (Wilson and Yoshimura 1994). Plant functional traits, defined as particular characteristics associated with plant morphology, growth, and life history, are assumed to be the most effective surrogates for plant strategies (Diaz and Cabido 2001; Thuiller et al. 2004). Of the physical environmental factors affecting ecological processes on the earth, climate is believed to be one of the decisive influences on plant distributions (Hamrick 2004; Woodward et al. 2004). Detecting relationships between trait variation and climate would thus provide a powerful means of disclosing the mechanisms underlying climatically driven species replacement (Cumming 2002; Kröpelin et al. 2008; Reichstein et al. 2014).

The relationship of species distributions to climatic conditions cannot well explain why species substitution occurs in nature; however, functional trait variation associated with species substitution does explain species substitution (Reichstein et al. 2014). Plant traits are determined by factors related to plant phylogeny and the environment (Givnish 1987; Dudley and Schmitt 1995; Desdevises et al. 2003). Plant phylogenetic background could, thus, inevitably confound the effect of climate on trait variation (Givnish 1987; Ackerly 2004; Watanabe et al. 2007). Partitioning the relative influences of climate and phylogeny on trait variation would provide insights that could help to reveal the mechanism underlying species turnover (Cavender‐Bares et al. 2009).

Spruces (*Picea* Dietr., Pinaceae), distributed in the Northern Hemisphere across Eurasia and North America, are dominant species in boreal forests and in cold‐temperate coniferous mountain forests (Spribille and Chytry 2002). The genus *Picea* includes approximately 35 species (Farjón 1990), nearly half of which (16 taxa, including seven endemic species) are naturally distributed in China (Fu et al. 1999), extending over a wide geographical range (23–53° N, 75–134° E) and exhibiting a steep climatic gradient (Zhou and Yang 1980; Li et al. 2012). Previous studies have characterized the overall influence of climate on spruce distribution (Li and Chou 1984; Weng and Jackson 2000), its growth (Box 1996; Koprowski 2013) and trait variation (Wright 1955; Luo et al. 2005). However, most of the previous studies of these topics in spruce have only focused on one or a few species. Little is known about the relationship between trait variation and species turnover for a wide array of spruce species at large scales and even less has been clarified about the relative roles of climate and phylogenetic background in driving morphometric trait variation. In addition, the spruce taxa in China show high variability in morphometric traits (Fu et al. 1999) and in phylogenetic affinities (Ran et al. 2006; Lockwood et al. 2013), which provides an opportunity to answer these questions.

Evidence from the fossil record (Miller 1989; LePage 2001) and from molecular phylogeny (Weng and Jackson 2000; Ran et al. 2006; Lockwood et al. 2013) indicates that *Picea* is an ancient genus whose current distribution is influenced by postglacial re‐expansion (Xu et al. 1980; Ran et al. 2006). In addition, morphological convergence among phylogenetically distinct species or populations (Lockwood et al. 2013) indicates a strong effect of climate selection. Accordingly, we hypothesized that the current climate should be the major determinant that affects trait variation, with phylogenetic background playing only a secondary role. In addition, if species turnover occurs because of differences among species in ecological amplitude, as has been well established, we would expect significant differences in morphometric traits among species.

In this study, we compiled a morphometric trait dataset for 10 spruce taxa that are naturally distributed in China. We systematically examined the phylogenetic and climatic components of trait variation as well as the relationship between trait variation and species turnover.

## Methods

### Study area

**Figure 1:**
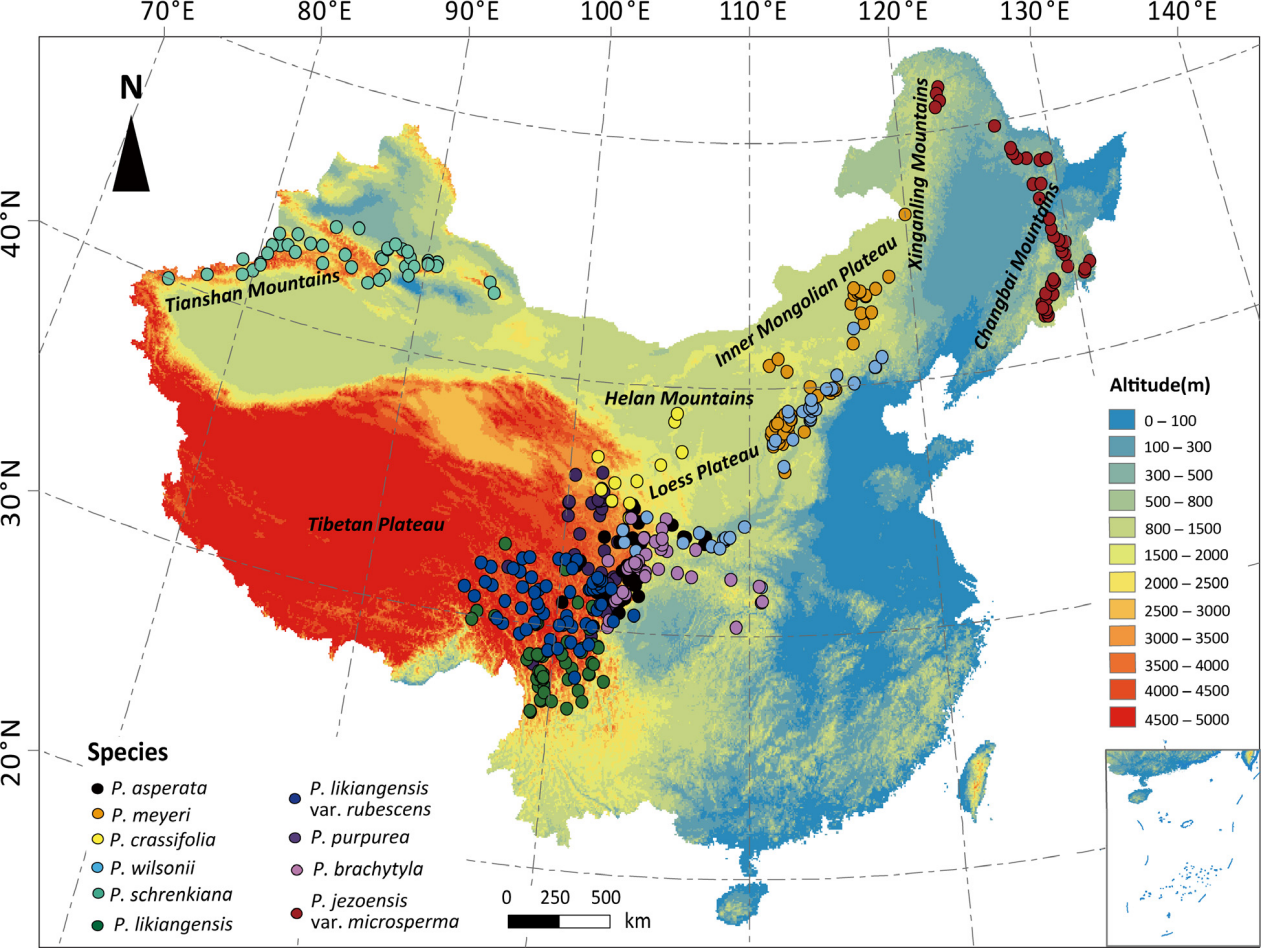
Locations of sampling sites for 10 spruce taxa in China.

The study area includes the main areas in which spruce forests occur in China (Fig. 1). The region encompasses a wide geographical range (23–53°N, 75–134°E) and altitudinal gradient (250–4300 m a.s.l.). The selected region represents a large climatic gradient with a mean annual temperature of −9.18 to 15.63°C and a mean annual precipitation of 159.49 to 1094.88 mm.

### Species

We selected 10 spruce taxa for analysis: *P. jezoensis* var. *microsperma*,* P. schrenkiana*,* P. crassifolia*,* P. meyeri*,* P. likiangensis*,* P. likiangensis* var*. rubescens*,* P. purpurea*,* P. brachytyla*,* P. wilsonii,* and *P. asperata*. These species are dominant components of natural cool‐temperate coniferous forests in the study area, with different species forming different communities distributed in different regions (Fig. 1). In addition, these selected species show clear patterns of spatial replacement either horizontally or vertically (Zhou and Yang 1980).

### Trait data

We measured nine morphometric traits of the needle leaves, seed cones, and seeds of the 10 spruce taxa (Table S1): leaf length (LL), leaf maximum width (LW), seed cone length (SCL), seed cone maximum diameter (SCD), seed scale length (SSL), seed scale maximum width (SSW), seed length (SL), seed wing length (SWL), and seed wing maximum width (SWW). These traits have been used in spruce classification (Fu et al. 1999) and have implications for characterizing spruce acclimation and adaptation to climate gradients (Givnish 1987; Eckenwalder 2009).

The morphometric traits were measured on the samples collected during our field surveys at 14 sites and on 2956 spruce specimens at 490 sites collected for the Herbarium of Institute of Botany, Chinese Academy of Sciences. To ensure comparability with the herbarium specimens, the samples collected during our field surveys were air‐dried for several days before measurement. The geographical coordinates of each site were accurately recorded during our field surveys. Needle leaves, seed cones, and seeds of the dominant spruce species were collected from at least five well‐grown trees, and five replicates were available for each trait at each site. Trait data based on the herbarium specimens were measured on at least five specimens of spruce at each site. Between 13 and 143 sites were sampled for morphometric traits of the 10 species (Table S1). We calculated the mean value for a given species at a given site for each trait, yielding a species (site) × trait matrix.

The sites identified by consulting the herbarium records were electronically revisited and resampled based on Google Earth to obtain precise coordinates. Thus, 504 sites with latitude, longitude, and altitude data (a geographical location × site matrix) and species presence/absence data (a species × site matrix) were available for this study.

### Phylogenetic data

We retrieved DNA sequences from NCBI GenBank (www.ncbi.nlm.nih.gov) to construct a phylogenetic tree of the 10 studied species (Figure S1). Our phylogenetic tree was constructed by Mega 6.0 software (Tamura et al. 2013) based on three plastid (trnL‐trnF, trn‐psbA, and trnS‐trnG) and two mitochondrial (nad5 intron 1 and nad1 intron 2) DNA sequences. These sequences were previously used by Lockwood et al. (2013).

### Climate data

The original climate data, recorded from 1971 to 2000, including monthly mean temperature, precipitation, and average monthly extreme high and low temperatures, were derived from 1814 meteorological stations across China (Chinese Central Meteorological Bureau, 2003, unpubl. data). The data were regridded to a 10′ latitude by 10′ longitude grid by the smoothing spline method (Hutchinson 1989). Nine bioclimatic variables were then calculated from the original climate data via the BIOME3 model (Haxeltine and Prentice 1996): mean annual precipitation, MAP; mean annual temperature, MAT; mean temperature of the coldest month, TCM; mean temperature of the warmest month, TWM; growing degree days on a 0°C and 5°C basis, GDD0 and GDD5, respectively; actual evapotranspiration, AET; potential evapotranspiration, PET; and aridity index (AET/PET, *α*). A climate variables × site matrix was then determined from the geographical location × site matrix and the climate dataset (Table S2).

### Data analyses

To determine whether the selected morphometric traits were phylogenetically conserved, we measured the phylogenetic signal with *K* statistics (Blomberg et al. 2003) using the R package “picante.” Phylogenetic signal was tested for statistical significance by comparing the variance of independent contrasts for each trait with the expected values calculated by shuffling the values for the taxa at the tips 999 times, based on the species × trait matrix and the phylogenetic tree of the 10 studied species. *K* > 1 indicates strong phylogenetic signal (Kraft and Ackerly 2010). Furthermore, we evaluated the proportions of trait variation explained by phylogeny and by climatic variables as well as their combined effect (Desdevises et al. 2003). This analysis was performed using a phylogenetic eigenvector regression (PVR) analysis (Diniz Filho et al. 2012) with the R package “PVR,” based on the species × trait matrix, a species × climate matrix, and the phylogenetic tree of the 10 studied species. The sample size in each analysis was 504. The PVR method modeled variation in a trait (**Y**) with phylogenetic eigenvectors (**E**) and climate variables (**V**), and **E** was extracted from phylogenetic distance matrix using a principal coordinates analysis (PCoA) after a double‐centered transformation. A forward variable selection procedure was used to eliminate the redundant variables with R package “packfor,” and **E** or **V** was then separately used as predictor variables in a regression for a given **Y** (Diniz Filho et al. 1998). In Table 1, the variations in the traits explained, respectively, by phylogeny and environment were represented by *R* ^2^ of these regressions, marked as “*a*” and “*c*”; a multiple regression on both **E** and **V** was done, and the *R* ^2^ represents the variation explained by the combining effect of phylogenetic and environment, which was marked as “*b*”; and the unexplained variation was marked as “*d*.”

**Table 1:** Coefficients of determination of partial regression models of spruce morphometric variables against phylogenetic and climatic components. *K*, phylogenetic signal; *P*, statistical significance of phylogenetic signal; *a*, variation explained by climate only (%); *b*, shared variation between climate and phylogeny (%); *c*, variation explained by phylogeny only (%); *d*, unexplained variation (%)

Traits	*K*	*P*	*a*	*b*	*c*	*d*
Needle length	0.35	0.103	65	18	12	4.8
Needle width	0.56	0.008	59	14	11	15
Seed cone length	0.38	0.082	83	11	3.8	3.2
Seed cone diameter	0.31	0.125	69	5.1	16	9.6
Seed scale length	0.19	0.405	39	26	8.1	26
Seed scale width	0.68	0.013	71	24	3.6	1.3
Seed wing length	0.45	0.039	45	36	4.8	15
Seed wing width	0.55	0.016	59	18	3.8	19
Seed length	0.31	0.180	70	6.9	11	12

To examine the relationship between climate and spruce distribution, we performed a canonical correspondence analysis (CCA) based on the climate × site matrix with the trait × site matrix. CCA is a weighted method, and the environmental data are reweighted at each permutation step using permuted weights and a pseudo‐*F* value was calculated as a measure of the significance of the overall analysis (Ter Braak 1987). In this case, the mean value per species calculated from all trait measurements from a given site was used as a response variable. The climatic variables were log‐transformed before analysis. To reduce the arch effect in the CCA, we performed forward selection with the Holm correction (a *P*‐value correction method) to eliminate redundant climatic variables. As a result, the variables TCM, MAP, TWM, and *α*, which were selected with the Holm correction, were considered in the subsequent analyses. We used an ordinary least squares (OLS) linear regression to detect the relationship between traits and climatic variables. A one‐sample test statistic was used to evaluate whether the trend observed within a species was significantly different from the overall trend across species (Warton et al. 2012).

The statistical analyses other than the CCA, which was run in the Canoco5 software tool (Šmilauer and Lepš 2014), were performed using the R 3.1.2 software environment for statistical computing and graphics (R Core Team, 2014).

## Results

### Trait variation and phylogeny

The estimated *K*‐values for the nine traits were less than 1. Specifically, the *K*‐values for the traits ranged between 0.19 and 0.68. The *K*‐values for four traits (i.e., needle width, seed scale width, seed wing length, and seed wing width) were significant at *P* < 0.05. A further PVR analysis showed that the predictive power (*R* ^2^) of the corresponding full models ranged from 0.88 to 0.98. The percentage of variation explained solely by phylogeny was very low, ranging from 3.6% to 16%, whereas the percentage of variation explained by climate was much higher, ranging from 39% to 83% (Table 1).

### Trait variation along climatic gradients revealed by CCA

**Figure 2:**
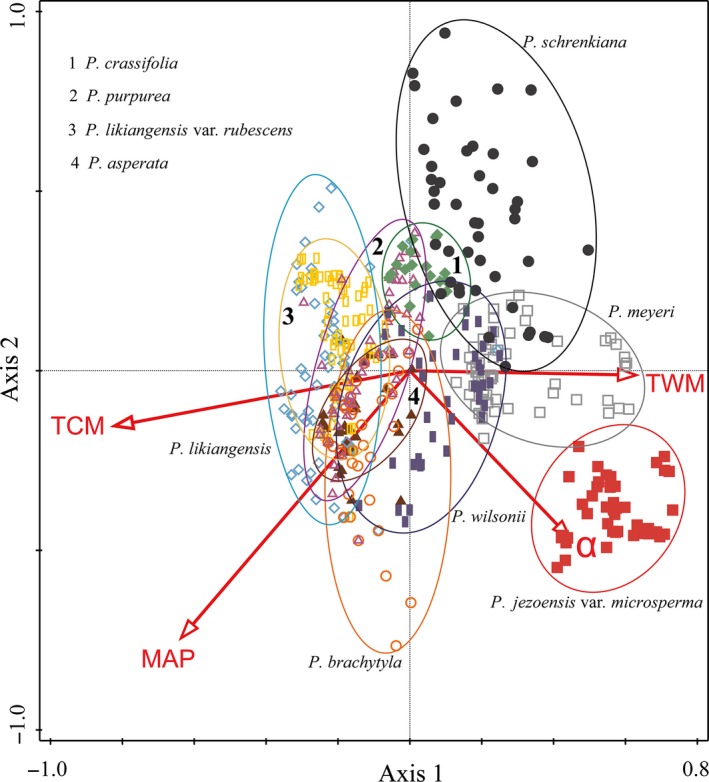
The relationship between morphometric traits of the 10 spruce taxa and climate factors as determined by a canonical correlation analysis (CCA). Each species is shown in a different shape and color to identify the ecological niche position occupied and is enclosed in an ellipse. The area of the ellipse represents the ecological amplitude of the species to some extent. The projection of the ellipse on a climatic axis represents the niche breadth of the species on this climatic gradient. TCM, mean air temperature of the coldest month; TWM, mean air temperature of the warmest month; MAP, mean annual precipitation; *α*, aridity index.

The first two CCA axes explained 76.47% of the variance in the trait–climate relationships (Table 2). The forward selection procedure indicated that TCM, MAP, TWM, and *α* could well represent the climate variables. The pseudo‐*F* values of the four climate variables were 72.45, 51.7, 59.52, and 24.48. The first CCA axis was determined largely by temperature and, to a lesser extent, by moisture. This gradient was characterized by decreasing TCM and MAP as well as by increasing TWM and *α*, representing a seasonality gradient varying from moderate wet and warm winters but cool summers (hereafter, cool climate) to dry and warm summers but cold winters (hereafter, warm climate). The second axis, however, was a moisture gradient determined by MAP and *α* with the wet end at the bottom and the dry end at the top of the CCA biplot. Along the temperature gradient from the warm‐climate habitats to the cool‐climate habitats during the growing season, *P. schrenkiana, P. meyeri,* and *P. jezoensis* var. *microsperma* were found at the warm end of the spectrum. As TCM increased, *P. wilsonii* and *P. meyeri* appeared. The other five species aggregated at the cool end of the spectrum, corresponding to further increases in TCM. Along the moisture gradient from wet to dry, *P. brachytyla* and *P. jezoensis* var. *microsperma* were present at the wet end, while *P. wilsonii*,* P. meyeri*,* P. purpurea*, and *P. likiangensis* tended to be present in climates with moderate moisture conditions. As MAP decreased further, *P. crassifolia* began to appear, and *P. schrenkiana* was present at the dry end of the gradient (Fig. 2).

**Table 2:** Summary of CCA of morphometric traits of the 10 spruce taxa in relation to key climate variables based on forward selection: mean temperature of the coldest month (TCM), mean annual precipitation (MAP), mean temperature of the warmest month (TWM), and aridity index (*α*)

	Climate variables	Axis 1	Axis 2	Axis 3
Correlation coefficients	TCM	−0.76	−0.11	−0.25
	TWM	0.58	−0.087	−0.36
	MAP	−0.58	−0.53	−0.087
	*α*	0.41	−0.34	−0.35
Eigenvalues		0.85	0.51	0.27
Explained variation (%)		47.68	28.79	14.94
Cumulative % variation of trait–climate relations		47.68	76.47	91.41
Significance (permutation test on all axes): *pseudo‐*F* = 2.5, *P* = 0.002				

*The pseudo‐*F* value is a measure of the significance of the overall analysis.

### Trait–climate relationships observed within and across species

TCM and MAP had relatively strong correlations with the first axis (−0.76 for TCM) and the second axis (−0.53 for MAP) of the CCA of the trait–climate relationship (Table 2). We therefore used these two variables in all subsequent OLS linear regression analyses.

**Figure 3:**
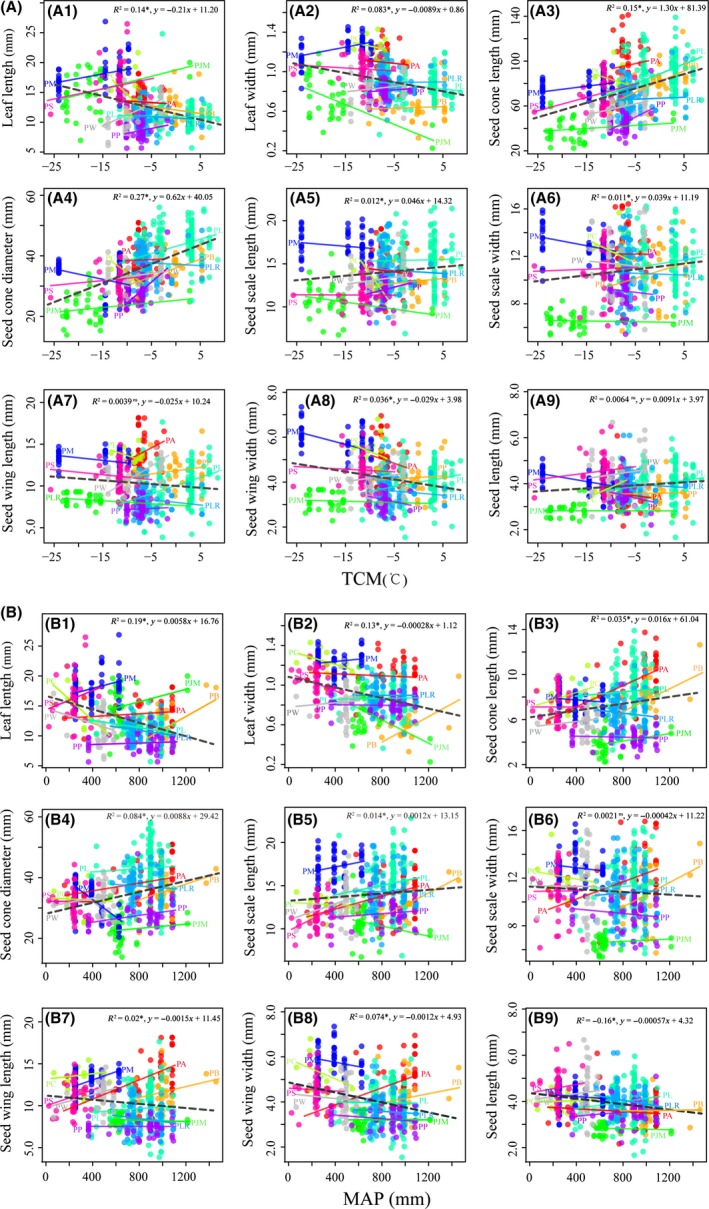
Relationship between each trait and temperature for the coldest month (TCM) and mean annual precipitation (MAP). The solid‐colored lines represent the regressions for individual species, and the black dotted line represents the regression for all species. *R*², slope, and intercept are displayed at the top of each figure. *Indicates that the regression relationship is significant (*P *<* *0.05). PA,* Picea asperata*; PM,* Picea meyeri*; PL,* Picea likiangensis*; PLR,* Picea likiangensis* var*. rubescens*; PB,* Picea brachytyla*; PP,* Picea purpurea*; PC,* Picea crassifolia*; PW,* Picea wilsonii*; PS *, Picea schrenkiana;* PJM,* Picea jezoensis* var. *microsperma*.

For all taxa taken together, LW and LL decreased with increasing TCM (Fig. 3A1, A2) and MAP (Fig. 3B1, B2), indicating that leaf size was smaller in wet‐cool climates than in dry‐warm climates. SCL and SCD increased with increasing TCM (Fig. 3A3, A4) and MAP (Fig. 3B3, B4), indicating that seed cone size was larger in wet‐cool climates than in dry‐warm climates. SSL and SSW tended to be loosely correlated with TCM (Fig. 3A5, A6) and MAP (Fig. 3B5, B6), as indicated by either small slopes of the regression lines or nonsignificant *R* ^2^ values (SSW‐MAP). Seed wing width (SWW) and length (SWL) decreased with increasing TCM (Fig. 3A7, A8) and MAP (Fig. 3B7, B8), suggesting that seed wing size was smaller in wet‐cool climates than in dry‐warm climates. SL decreased with increasing MAP (Fig. 3B9) but not with TCM (Fig. 3A9), indicating that seeds were shorter in wet climates than in dry climates.

Further analyses of the trends in trait variation relative to climate, as observed within a species, were not necessarily consistent with those observed across species (Fig. 3; Table S3). Detailed descriptions of trait variation with climate within species are presented in Appendix S1.

## Discussion

### Trait variation is largely determined by climate rather than by plant phylogeny

Plant phylogenetic background and abiotic, biotic environments are assumed to be the two potential determinants that explain plant trait variation (Givnish 1987; Ackerly 2004; Watanabe et al. 2007). Our results showed that variation in the morphometric traits of the studied spruces is largely determined by climate rather than by plant phylogeny. In fact, morphological divergences among spruce species do not necessarily match their phylogenetic divergences. For example, spruce species worldwide do not form a phylogenetically defined clade (Ran et al. 2006; Lockwood et al. 2013) corresponding to any of the morphologically defined sections (Taylor 1993; Fu et al. 1999). Similar situations are even observed on a within‐species scale. Specifically, at least three spruces, namely *P. abies* in Europe, *P. brachytyla* in China, and *P. engelmannii* in North America, are not monophyletic, suggesting that the populations within a species are morphologically similar but could have originated from different ancestors (Lockwood et al. 2013). Although nonmonophyly of species does not necessarily indicate convergent evolution if there is introgression via hybridization, our findings indicate a strong selective effect of climate on species or populations that currently occur in the same climatic domain but have different phylogenetic backgrounds. Therefore, parallel evolution, that is, the repeated appearance of similar characteristics that occur among distantly related species (Went 1971; Schluter et al. 2004; Orr 2005), is quite common in spruce species (See Figure S2). These findings are in line with evidence from the fossil record (Xu et al. 1980; Miller 1989; LePage 2001) and molecular phylogeny studies (Weng and Jackson 2000; Ran et al. 2006; Lockwood et al. 2013) that shows that the diversification of all living spruce species was most likely finished by the end of the Tertiary (Pliocene), whereas the present distribution was influenced by postglacial re‐expansion.

Adaptive evolution often decreases phylogenetic signal strength. Therefore, a relatively small proportion of trait variation explained by plant phylogeny may indicate substantial adaptive evolution in spruce. For spruce species, such qualitative morphological characters as leaf cross sections, the position of stomata on the leaf surface, and the shape of the seed scales are undoubtedly adaptive traits. In addition, as revealed by a common‐garden study, Norway spruce populations from cold mountain environments show several adaptive features in plant physiological traits (Oleksyn et al. 1998). Of the morphometric traits measured in this study, the widths of the needle leaf, seed scale, and seed wing showed lower but significant phylogenetic signal, indicating that such trait values and trait similarity are more closely related to phylogenetic distance. Although the significant relationship between the morphometric traits and climatic variables is quite obvious, further studies are needed to clarify whether the observed trait variations are a result of acclimation or adaptation.

### Spruce ecological amplitudes tend to be wider for moisture compared with for temperature

The range of environmental conditions under which spruce species are observed to occur, that is, the ecological amplitudes of spruce species, are very narrow. The growth and persistence of spruce species are primarily favored by environmental/climatically driven light shading conditions and/or low light levels and by wet‐cool climates (Zhou and Yang 1980). In addition, temperature tends to be one of the decisive factors that influence the distribution, growth, and reproduction of spruces, and moisture conditions tend to be the second most influential factor (Gordon and Sirois 1992; Spribille and Chytry 2002; Meunier et al. 2007). Consistent with this principle, our trait‐based ordination showed that spruce distribution can be explained first by a temperature gradient (47.68%) and then by a moisture gradient (28.79%). Our results further showed that most of the species grew over a wider range along the moisture gradient than along the temperature gradient, although *P. meyeri* and *P. jezoensis* var. *microsperma* were exceptions to this pattern.

Moderate summer temperatures are critical for spruce seed maturation and viability, demography, and radial growth (Gordon and Sirois 1992; Kullman 1996; Johnsen et al. 2005). For example, approximately 800–940 growing degree days >5°C is viewed as the threshold for seed maturation in *Picea mariana* (Meunier et al. 2007), whereas the temperature in the coldest month is related to the dynamics of permafrost and ground frost, two environmental factors that are most likely influential in winter desiccation in spruce forests (Kullman and Engelmark 1997). In addition, low soil temperatures suppress soil microorganisms and nutrient mineralization (Šmilauer and Lepš 2014). Low winter temperatures are particularly harmful for spruce seedlings (Owens et al. 1993). Therefore, the optimal temperature conditions for spruce cannot be characterized by any single thermal variable but by a combination of summer and winter temperatures. In this case, we identified an abiotic and biotic varying from (1) warm winters, cool summers to (2) cold winters, hot summers. Most of the spruce species in question tended to be favored by a climate with moderate wet and thermal conditions, that is, warm winters but cool summers and with fairly high precipitation.

Once the appropriate temperature conditions are met, moisture becomes the key factor influencing spruce growth (Cleve et al. 1981; Wang et al. 2002). Our results showed that at a given temperature, most of the spruces showed a broad ecological amplitude on the moisture gradient. These characteristics result in frequent replacement between species along the temperature gradient but less frequent replacement along the moisture gradient (Fig. 2). This finding is consistent with the predictions of the species distribution hypothesis (Spalding 1890; MacArthur 1972; Pulliam 2000).

*Picea schrenkiana*,* P. meyeri,* and *P. jezoensis* var. *microsperma* tended to represent exceptions to the abovementioned pattern. These three species had a wide amplitude on both gradients but demonstrated distinct between‐species replacement. In fact, of the factors that influence plant distribution, geographical barriers may substantially mediate the climatically driven pattern (Just 1947). Unlike the other seven species, these three species are geographically isolated by geographical barriers such as deserts, plateaus, and plains (Fig. 1). The factors associated with geological history might have played a major role in shaping the current distributions of the studied spruces. The other seven species are distributed along the eastern margin of the Tibetan Plateau. Their distribution ranges even overlap somewhat (Fig. 1), which could represent a climatically driven pattern.

It is worth noting that these ecological amplitudes are measured relative to the range of climates in which these spruces are observed to occur. It is not relative to the total climatic space in the world or even to the total climatic space to which these spruce species could potentially occur. That could, although not necessarily, result in different values for relative amplitudes of temperature and precipitation niches for individual species.

### Trait variation as a proxy of plant ecological amplitude captures species turnover driven by climate

Particularly salient among the studied taxa was a tendency for needle leaf and seed size to decrease, whereas, in contrast, seed cone and seed scale size tended to increase as the climate varied from dry warm to wet cool. These overall trends, however, are not necessarily consistent with those observed for individual spruce species in this case as well as in previous studies of *P. mariana* (Khalil 1984), *P. asperata* (Luo et al. 2005), and *P. crassifolia* (Wang and Li 2008). The underlying mechanism is most likely species specific. In detail, to achieve survival in a given environment, different species may adopt different strategies, as predicted by the niche partitioning hypothesis (MacArthur 1968; Ackerly and Cornwell 2007; Honorio Coronado et al. 2015). In terms of trait variation within given environments, the functional, taxonomic, and phylogenetic background of species that show similar responses may differ (Cornwell and Ackerly 2009; Wang et al. 2015). Consistent with this principle, we found that the trait–climate relationships were quite species specific for the 10 spruce taxa. In addition, it should be intriguing to know whether or not there is a relationship between the level of phylogenetic signal for traits and how well species trends match the overall trend for the same traits. If this is the case, it would be interesting if the traits for which species match well the overall trend show the lowest phylogenetic signal. Such traits might have the highest phenotypic plasticity (and lack evolutionary constraint), which could lead to good matching of overall means and reduced phylogenetic signal. Given the relative low phylogenetic signal for traits and the less patterning intraspecific trends of trait variation with climatic gradient in this case, however, such a relationship between the level of phylogenetic signal for traits and how well species trends match the overall trend for the same traits is less evident. Further studies are needed to clarify this issue.

Our OLS regression analysis showed that species tended to successively replace one another as the environment became wetter and warmer in winters but cooler in summers, falling along a single overall trend describing variation in the traits of spruce in relationship to climate. Leaf size is relevant to light interception and plant photosynthesis (Wright and Westoby 2002; Ackerly 2004). The size of the seed cone, seed scale, and seed is related to seed formation, protection, and dispersal (Tomlinson and Takaso 2002). Of the morphometric traits selected in this case, leaf size and seed size tended to be smaller, whereas seed cone size tended to be larger as the climate became wetter and warmer in winter but cooler in summers, suggesting plausible tradeoffs between plant growth and reproduction in the face of changing climate. Although the physiological or ecological implications of trait variation with climate definitely need to be further clarified, our findings indicate that the morphometric traits carry strong climatic signals and, furthermore, that between‐species trait variation is highly consistent with species replacement, which thus should be included among the mechanisms underlying the observed turnover among the 10 spruce taxa.

## Conflict of Interest

None declared.

## Supporting information


**Table S1.** Nine morphometric traits measured for each of the 10 spruce taxa. For each trait, mean ± standard deviations marked with different letters indicate a significant between‐species difference (Tukey test, *P* < 0.05).
**Table S2.** Mean ± standard deviation of geographical locations (LAN, latitude; LON, longitude; AL, altitude) and climatic variables for the 10 spruce taxa. MAT, mean annual air temperature; TCM, mean temperature of the coldest month; TWM, mean temperature of the warmest month; GDD5, growing degree days on a 5°C basis; GDD0, growing degree days on a 0°C basis; MAP, mean annual precipitation; AET, actual evapotranspiration; PET, potential evapotranspiration; *α* (AET/PET), aridity index.
**Table S3.** Results of ordinary least squares (OLS) regression analysis for the trait–climate relationships at the intraspecific scale. Climatic variables are: TCM (mean temperature of the coldest month), TWM (mean temperature of the warmest month), mean annual precipitation (MAP) and aridity index (*α*). In the *R*² column, *indicates that the regression relationship is significant at *P *<* *0.05. In the slope column, *indicates that the slope of the regression line is significantly different from the overall slope (*P *<* *0.05).
**Figure S1.** A phylogenetic tree for the 10 spruce taxa.
**Figure S2.** Contmap of the mean values of spruce traits at each order. LL, Leaf length (mm); LW, Leaf width (mm); SCL, Seed cone length (mm); SCD, Seed cone diameter (mm); SSL, Seed scale length (mm); SCW, Seed scale width (mm); SWL, Seed wing length (mm); SWW, Seed wing width (mm); and SL, Seed length (mm).
