# Supplementary material for: Morphometric traits capture the climatically driven species turnover of 10 spruce taxa across China
Source: Ecol Evol. 2016 Jan 25;6(4):1203–13. doi: 10.1002/ece3.1971 (PMC4725448; doi:10.1002/ece3.1971)
Supplement: Supplementary file 1 — Table S1. Nine morphometric traits measured for each of the 10 spruce taxa. For each trait, mean ± standard deviations marked with different letters indicate a significant between‐species difference (Tukey test, P < 0.05). Table S2. Mean ± standard deviation of geographical locations (LAN, latitude; LON, longitude; AL, altitude) and climatic variables for the 10 spruce taxa. MAT, mean annual air temperature; TCM, mean temperature of the coldest month; TWM, mean temperature of the warmest month; GDD5, growing degree days on a 5°C basis; GDD0, growing degree days on a 0°C basis; MAP, mean annual precipitation; AET, actual evapotranspiration; PET, potential evapotranspiration; α (AET/PET), aridity index. Table S3. Results of ordinary least squares (OLS) regression analysis for the trait–climate relationships at the intraspecific scale. Climatic variables are: TCM (mean temperature of the coldest month), TWM (mean temperature of the warmest month), mean annual precipitation (MAP) and aridity index (α). In the R² column, *indicates that the regression relationship is significant at P < 0.05. In the slope column, *indicates that the slope of the regression line is significantly different from the overall slope (P < 0.05). Figure S1. A phylogenetic tree for the 10 spruce taxa. Figure S2. Contmap of the mean values of spruce traits at each order. LL, Leaf length (mm); LW, Leaf width (mm); SCL, Seed cone length (mm); SCD, Seed cone diameter (mm); SSL, Seed scale length (mm); SCW, Seed scale width (mm); SWL, Seed wing length (mm); SWW, Seed wing width (mm); and SL, Seed length (mm). [file ECE3-6-1203-s001.docx]

**Appendix**

**Table S1.** 9 morphometric traits measured for each of the 10 spruce taxa. For each trait, mean ± standard deviations marked with different letters indicate a significant between-species difference (Tukey test, p < 0.05).

| **Species** | **Mean (mm) ± Standard deviation** | | | | | | | | | |
| --- | --- | --- | --- | --- | --- | --- | --- | --- | --- | --- |
|  | *n* | Needle length | Needle width | Cone length | Cone diameter | Seed scale length | Seed scale width | Seed wing length | Seed wing width | Seed length |
| *Picea asperata* | 49 | 136.44±2.46^de^ | 1.09 ±0.15^ef^ | 95.96±22.31^f^ | 38.58±7.05^d^ | 14.27±2.62^de^ | 12.14±2.38^cd^ | 3.56±0.67^e^ | 13.88±2.94^e^ | 4.95±0.95^b^ |
| *Picea meyeri* | 56 | 178.21±2.63^g^ | 1.23 ±0.12^g^ | 75.76±12.77^de^ | 32.49±5.77^c^ | 17.09±1.64^f^ | 13.39±4.19^d^ | 4.18±0.48^de^ | 13.03±1.30^f^ | 5.73±0.74^cd^ |
| *Picea likiangensis* | 143 | 109.85±2.03^b^ | 0.90 ±0.38^cd^ | 84.84±20.65^e^ | 42.83±7.37^f^ | 15.18±2.88^e^ | 11.31±2.28^c^ | 4.06±0.97^b^ | 9.80±2.44^bc^ | 3.96±0.95^c^ |
| *Picea likiangensis* var. *rubescens* | 76 | 107.77±1.68^b^ | 0.90 ±0.12^d^ | 64.48±16.05^bc^ | 36.93±5.24^de^ | 14.22±2.86^de^ | 10.77±2.20^c^ | 3.98±0.57^a^ | 8.40±1.67^ab^ | 3.62±0.66^bc^ |
| *Picea brachytyla* | 28 | 121.92±2.62^bcd^ | 0.64 ±0.17^a^ | 80.08±16.70^cde^ | 35.44±5.02^cd^ | 12.90±1.46^bcd^ | 10.70±2.46^bcd^ | 3.62±0.55^cd^ | 11.72±1.88^cd^ | 4.20±0.90^b^ |
| *Picea purpurea* | 47 | 87.54 ±1.58^a^ | 0.81±0.09^bc^ | 43.88±9.69^a^ | 28.02±4.52^b^ | 11.78±1.52^abc^ | 9.06±1.36^b^ | 3.55±0.51^a^ | 7.39±0.91^a^ | 3.30±0.45^b^ |
| *Picea crassifolia* | 13 | 144.06±4.14^def^ | 1.23±0.12^fg^ | 81.07±12.66^ef^ | 32.70±4.69^bcd^ | 12.76±1.55^abcd^ | 12.09±1.63^cd^ | 3.99±0.39^ef^ | 13.3±81.32^ef^ | 5.24±0.78^bcd^ |
| *Picea wilsonii* | 34 | 117.11±4.11^bc^ | 0.83±0. 18^b^ | 58.61±10.96^b^ | 33.61±4.33^cde^ | 13.28±2.19^cd^ | 11.32±2.90^c^ | 4.40±1.00^cd^ | 11.35±1.54^cd^ | 4.20±0.75^cd^ |
| *Picea schrenkiana* | 39 | 162.59±3.64^fg^ | 1.02±0.14^de^ | 71.08±14.45^cd^ | 32.07±4.55^bc^ | 11.40±1.68^a^ | 10.96±2.11^c^ | 4.53±0.75^de^ | 11.1±21.78^de^ | 4.50±0.69^de^ |
| *Picea jezoensis* var. *microsperma* | 33 | 150.18 ±3.29^ef^ | 0.73±0.22^ab^ | 38.53±10.54^a^ | 22.68±4.86^a^ | 10.79±0.34^a^ | 6.59±1.97^a^ | 2.85±0.36^a^ | 8.43±0.63^a^ | 3.17±0.37^a^ |

**Table S2.** Mean ± standard deviation of geographical locations (LAN, latitude; LON, longitude; AL, altitude) and climatic variables for the 10 spruce taxa. MAT, mean annual air temperature; TCM, mean temperature of the coldest month; TWM, mean temperature of the warmest month; GDD5, growing degree days on a 5˚ C basis; GDD0, growing degree days on a 0˚ C basis; MAP, mean annual precipitation; AET, actual evapotranspiration; PET, potential evapotranspiration; α (AET/PET), aridity index.

| **Species** | **LAN** | **LON** | **MAT** | **TCM** | **TWN** | **GDD5** | **GDD0** | **MAP** | **SM** | **AET** | **PET** | **α** |
| --- | --- | --- | --- | --- | --- | --- | --- | --- | --- | --- | --- | --- |
| *P. asperata* | 30.05-37.01 | 100.31-107.69 | 2.18±0.34 | -6.41±0.27 | 10.09±0.48 | 567.36±67.52 | 1434.42±93.21 | 901.81±22.55 | 88.87±1.41 | 373.60±28.54 | 461.8±20.94 | 0.32±0.01 |
| *P. meyeri* | 36.56-46.65 | 111.27-120.09 | 2.81±0.26 | -14.72±0.5 | 17.99±0.2 | 1460.29±36.49 | 2445.94±44.42 | 439.24±11.42 | 65.85±1.91 | 647.91±7.32 | 374.96±8.35 | 0.57±0.01 |
| *P. likiangensis* | 25.05-36.62 | 5.77-105.88 | 6.36±0.31 | -1.04±0.36 | 12.82±0.29 | 1243.71±68.03 | 2561.95±94.73 | 856.68±10.73 | 84.37±0.56 | 474.29±15.51 | 705.01±15.94 | 0.48±0.01 |
| *P. likiangensis* var*. rubescens* | 27.93-32.71 | 95.04-104.08 | 2.34±0.26 | -6.13±0.26 | 9.62±0.28 | 522.93±56.91 | 1454.58±75.51 | 788.51±11.5 | 89.67±0.22 | 440.61±18.16 | 498.29±19.88 | 0.37±0.01 |
| *P. brachytyla* | 28.26-34.60 | 101.99-110.75 | 6.88±0.64 | -2.35±0.56 | 15.47±0.74 | 1576.85±144.16 | 2813.47±190.9 | 950.63±24.11 | 90.27±1.53 | 556.23±27.04 | 497.69±29.25 | 0.5±0.020 |
| *P. purpurea* | 27.45-36.31 | 97.28-104.35 | 1.09±0.24 | -8.29±0.33 | 9.37±0.31 | 407.78±40.79 | 1238.11±59.74 | 778.04±24.66 | 88.68±1.19 | 395.08±25.9 | 457.15±23.2 | 0.35±0.011 |
| *P. crassifolia* | 34.04-40.91 | 98.68-111.16 | 2.2±0.54 | -11.2±0.48 | 13.98±0.69 | 1001.4±103.02 | 1921.79±129.44 | 411.21±21.63 | 65.3±4.11 | 605.09±23.33 | 344.27±32.87 | 0.38±0.022 |
| *P. wilsonii* | 30.83-42.24 | 101.31-117.94 | 6.82±0.45 | -7.53±0.48 | 19.38±0.56 | 1972.16±104.68 | 3121.64±125.85 | 592.57±20.95 | 77.24±1.61 | 706.39±10.15 | 407.78±13.84 | 0.55±0.011 |
| *Picea schrenkiana* | 37.02-45.94 | 75.16-94.49 | 3.47±0.54 | -13.59±0.45 | 18.21±0.69 | 1652.34±105.96 | 2620.28±128.67 | 269.44±11.4 | 30.03±2.5 | 639.03±12.94 | 232.47±14.02 | 0.35±0.024 |
| *P. jezoensis* var*. microsperma* | 41.96-52.49 | 124.00-131.24 | 1.87±0.24 | -19.53±0.37 | 19.98±0.18 | 1631.19±35.42 | 2589.02±42.36 | 637.9±9.4 | 87.67±0.21 | 484.39±12.43 | 456.56±13.9 | 0.68±0.013 |

**Table S3.** Mean ± standard deviation of geographical locations (LAN, latitude; LON, longitude; AL, altitude) and climatic variables for the 10 spruce taxa. MAT, mean annual air temperature; TCM, mean temperature of the coldest month; TWM, mean temperature of the warmest month; GDD5, growing degree days on a 5˚ C basis; GDD0, growing degree days on a 0˚ C basis; MAP, mean annual precipitation; AET, actual evapotranspiration; PET, potential evapotranspiration; α (AET/PET), aridity index.

|  |  | *Sect. Picea* | | | | | | | | | | | | | | |
| --- | --- | --- | --- | --- | --- | --- | --- | --- | --- | --- | --- | --- | --- | --- | --- | --- |
|  | Species | *Picea asperata* | | | *Picea meyeri* | | | *Picea crassifolia* | | | *Picea wilsonii* | | | *Picea schrenkiana* | | |
| Climate factors | *n* | 50 | | | 56 | | | 11 | | | 34 | | | 39 | | |
|  | Trait | R² | Slope | Intercept | R² | Slope | Intercept | R² | Slope | Intercept | R² | Slope | Intercept | R² | Slope | Intercept |
| MAP | Leaf length | 0.0033 | 0.00065* | 13.04 | 0.12* | 0.00064* | 15.29 | 0.34* | -0.017 | 19.57 | 0.022 | -0.0062 | 14.66 | 0.029 | 0.00079 | 14.38 |
|  | Leaf width | 0.0039 | -0.00042* | 1.13 | 0.0058 | 0.000066* | 1.21 | 0.15 | -0.0030 | 1.32 | 0.0067 | 0.0015 | 0.76 | 0.065 | -0.00047 | 1.13 |
|  | Seed cone length | 0.21* | 0.048* | 52.25 | 0.0168 | -0.0094* | 80.55 | 0.13 | 0.032 | 71.46 | 0.0098 | 0.011 | 53.44 | 0.00026 | -0.003 | 71.15 |
|  | Seed cone diameter | 0.038 | 0.0063 | 32.75 | 0.504* | -0.029* | 44.18 | 0.029 | -0.0018 | 33.24 | 0.019 | 0.0061 | 30.73 | 0.00015 | -0.0071 | 32.24 |
|  | Seed scale length | 0.1046* | 0.0039 | 10.68 | 0.04 | 0.0024 | 16.15 | 0.004 | -0.00072 | 12.97 | 0.045 | 0.0048 | 11.02 | 0.09 | 0.0065 | 9.8 |
|  | Seed scale width | 0.11* | 0.0036* | 8.824 | 0.0024 | -0.00045 | 13.03 | 0.024 | -0.0017 | 12.62 | 0.00021 | 0.00013 | 11.25 | 0.00087 | 0.0008 | 10.94 |
|  | Seed wing length | 0.17* | 0.0056 | 8.71 | 0.19* | 0.0041* | 11.42 | 0.0025 | 0.00046 | 13.24 | 0.032 | 0.0028 | 10.01 | 0.045 | 0.0048 | 9.98 |
|  | Seed wing width | 0.21* | 0.0020* | 3.14 | 0.013 | -0.00061 | 5.97 | 0.14 | -0.02 | 5.85 | 0.0025 | -0.00039 | 4.38 | 0.001 | -0.00028 | 4.57 |
|  | Seed length | 0.0025 | -0.00015 | 3.71 | 0.01 | -0.00044 | 4.35 | 0.00022 | 0.00039 | 3.98 | 0.0058 | 0.00078 | 4.04 | 0.00699 | 0.00081 | 4.34 |
| TCM | Leaf length | 0.0011 | -0.049 | 13.29 | 0.12* | 0.16* | 20.29 | 0.084 | -0.02 | 9.14 | 0.15* | 0.37* | 14.64 | 0.014 | 0.15 | 18.01 |
|  | Leaf width | 0.00055 | -0.022 | 1.07 | 0.23* | 0.11* | 1.39 | 0.040 | -0.012 | 1.14 | 0.031 | 0.0071* | 0.89 | 0.0035 | -0.0029 | 0.99 |
|  | Seed cone length | 0.0039 | 0.87 | 102.12 | 0.065* | 0.45* | 83.84 | 0.039 | -1.29 | 69.96 | 0.098* | 0.79 | 64.90 | 0.06 | 1.21 | 85.41 |
|  | Seed cone diameter | 0.0013 | -0.0051 | 38.55 | 0.098* | -0.31 | 27.57 | 0.24 | -1.18* | 22.55 | 0.026 | 0.16* | 34.88 | 0.007 | 0.13 | 33.65 |
|  | Seed scale length | 0.00075 | -0.015 | 14.17 | 0.02 | -0.045* | 16.38 | 0.11 | -0.27 | 10.45 | 0.04 | 0.11 | 14.11 | 0.00034 | 0.0033 | 11.11 |
|  | Seed scale width | 0.00023 | -0.023 | 12.12 | 0.13 | -0.081* | 11.58 | 0.03 | -0.16 | 10.75 | 0.0048 | -0.047 | 10.95 | 0.0008 | 0.021 | 11.21 |
|  | Seed wing length | 0.031 | 0.32 | 16.14 | 0.047 | -0.049 | 12.26 | 0.09 | -0.21 | 11.61 | 0.08 | 0.11* | 12.14 | 0.0077 | -0.053 | 10.49 |
|  | Seed wing width | 0.015 | -0.072 | 4.44 | 0.21* | -0.058* | 4.83 | 0.05 | -0.095 | 4.43 | 0.0089 | 0.016 | 4.33 | 0.001 | -0.078 | 4.41 |
|  | Seed length | 0.009 | -0.041 | 3.27 | 0.12* | -0.028* | 3.73 | 0.2 | 0.081 | 4.76 | 0.01 | 0.025 | 4.60 | 0.011 | 0.026 | 4.85 |
| TWM | Leaf length | 0.024 | -0.96 | 149.22 | 0.065* | 2.61 | 131.1 | 0.066 | -3.94 | 213 | 0.42** | 5.07** | -6.49 | 0.069 | -0.71 | 185.38 |
|  | Leaf width | 0.0052 | 0.0029 | 1.07 | 0.084* | -0.016* | 1.51 | 0.14 | -0.015 | 1.49 | 0.24** | 0.022** | 0.34 | 0.0098 | -0.005 | 1.06 |
|  | Seed cone length | 0.16** | -2.38** | 117.29 | 0.0082 | -0.41 | 84.02 | 0.0059 | 0.36 | 74.35 | 0.096 | 0.98* | 35.985 | 0.16** | -0.77 | 87.85 |
|  | Seed cone diameter | 0.021 | -0.28 | 41.12 | 0.099* | 0.81** | 18.16 | 0.050 | 0.38 | 35.73 | 0.0029 | -0.067 | 35.35 | 0.19** | -0.23 | 36.66 |
|  | Seed scale length | 0.12* | -0.23* | 16.30 | 0.042 | -0.15 | 19.74 | 0.011 | -0.055 | 13.57 | 0.014 | 0.079 | 11.62 | 0.17* | -0.096 | 13.81 |
|  | Seed scale width | 0.11* | -0.21* | 14.02 | 0.0069 | -0.048 | 13.69 | 0.12 | 0.21 | 8.43 | 0.050 | -0.21 | 16.08 | 0.019 | -0.061 | 7.78 |
|  | Seed wing length | 0.085 | -0.23* | 15.96 | 0.038 | -0.12 | 15.05 | 0.21 | -0.22 | 17.24 | 0.0012 | -0.016 | 11.79 | 0.072 | 0.056 | 9.67 |
|  | Seed wing width | 0.012 | -0.12** | 6.02 | 0.0020 | 0.0014 | 5.73 | 0.028 | 0.048 | 4.36 | 0.00085 | 0.0024 | 4.03 | 0.023 | 0.0090 | 4.16 |
|  | Seed length | 0.0031 | 0.0033 | 3.54 | 0.0097 | 0.021 | 3.81 | 0.023 | 0.021 | 3.65 | 0.0079 | 0.026 | 3.68 | 0.021 | -0.013 | 4.80 |
| α | Leaf length | 0.0088 | -0.059 | 13.8 | 0.0048* | 0.14 | 17.09 | 0.26 | -10.27* | 20.01 | 0.095 | -9.58* | 17.11 | 0.13* | 12.03* | 12.58 |
|  | Leaf width | 0.019 | 0.16* | 1.04 | 0.14* | -0.34 | 1.47 | 0.078 | -0.24 | 1.32 | 0.0024 | -0.066 | 0.87 | 0.034 | -0.24 | 1.09 |
|  | Seed cone length | 0.076* | -49.17* | 109.66 | 0.095* | -23.80 | 89.36 | 0.026 | 15.72 | 75.73 | 0.0047 | -5.31 | 61.84 | 0.018 | 17.92 | 65.61 |
|  | Seed cone diameter | 0.018 | -7.69 | 40.72 | 0.07* | -11.09 | 38.33 | 0.0011 | 0.12 | 32.66 | 0.0094 | 3.18 | 31.81 | 0.0099 | 4.18 | 30.79 |
|  | Seed scale length | 0.083* | -5.31* | 15.75 | 0.039 | 2.52 | 15.76 | 0.0013 | 0.14 | 12.71 | 0.025 | 2.61 | 11.81 | 0.098* | 4.8 | 9.94 |
|  | Seed scale width | 0.089* | -5.39 | 13.64 | 0.034 | 1.78* | 11.91 | 0.035 | -2.37 | 12.89 | 0.0028 | 1.05 | 10.73 | 0.0028 | 1.04 | 10.65 |
|  | Seed wing length | 0.12* | -7.64* | 16.01 | 0.19* | 4.29* | 10.78 | 0.0099 | 1.01 | 13.04 | 0.0096 | 0.36 | 11.15 | 0.12* | 5.23* | 9.53 |
|  | Seed wing width | 0.26* | -3.89* | 6.03 | 0.046 | 1.22* | 5.09 | 0.22 | -2.71 | 6.16 | 0.0082 | -0.52 | 4.4.9 | 0.013 | 0.73 | 4.28 |
|  | Seed length | 1.01 | 0.37 | 3.46 | 0.026 | 0.60 | 3.86 | 0.0057 | -0.701 | 4.01 | 0.0049 | 0.15 | 4.32 | 0.083* | 1.74 | 4.01 |

|  |  | *Sect. Casicta* | | | | | | | | | *Sect. Omorica* | | | | | |
| --- | --- | --- | --- | --- | --- | --- | --- | --- | --- | --- | --- | --- | --- | --- | --- | --- |
|  | Species | *Picea purpurea* | | | *Picea likiangensis* | | | *Picea likiangensis* var. *rubescens* | | | *Picea brachytyla* | | | *Picea jezoensis* var. *microsperma* | | |
| Climate factors | *n* | 47 | | | 143 | | | 76 | | | 28 | | | 34 | | |
|  | Trait | R² | Slope | Intercept | R² | Slope | Intercept | R² | Slope | Intercept | R² | Slope | Intercept | R² | Slope | Intercept |
| MAP | Leaf length | 0.00047 | 0.00016* | 8.62 | 0.0358* | -0.0030* | 13.56 | 0.0413* | -0.0026* | 12.91 | 0.20* | 0.0083* | 3.74 | 0.055 | 0.0051* | 11.6 |
|  | Leaf width | 0.00035 | -0.000075* | 0.81 | 0.00016 | 0.00015* | 0.85 | 0.00046 | -0.00021* | 0.91 | 0.12 | 0.00043* | 0.21 | 0.16 | -0.0058 | 1.11 |
|  | Seed cone length | 0.00063 | -0.0011* | 44.79 | 0.0032 | 0.0090 | 77.03 | 0.010 | -0.011* | 74.46 | 0.217* | 0.056* | 23. 74 | 0.052 | 0.016 | 27.88 |
|  | Seed cone diameter | 0.055 | 0.0048 | 24.10 | 0.0016 | 0.00225 | 40.87 | 0.0032 | -0.0023* | 38.78 | 0.115 | 0.012 | 23.12 | 0.013 | 0.0037 | 20.20 |
|  | Seed scale length | 0.0211 | 0.0010 | 10.96 | 0.013 | 0.0025 | 12.98 | 0.00017 | -0.0026 | 14.57 | 0.39* | 0.0065* | 6.3 | 0.04 | -0.0027 | 12.55 |
|  | Seed scale width | 0.031 | -0.0011 | 9.95 | 0.0027 | 0.00089 | 10.52 | 0.0014 | 0.00062 | 10.26 | 0.085 | 0.00515 | 5.5 | 0.0081 | 0.00041 | 6.32 |
|  | Seed wing length | 0.00066 | 0.00011 | 7.3 | 0.0026 | 0.00095 | 8.97 | 0.025 | -0.0020 | 10.05 | 0.047 | 0.0029 | 8.78 | 0.013 | -0.00049 | 8.75 |
|  | Seed wing width | 0.042 | -0.00042* | 3.64 | 0.00038 | -0.00013 | 4.06 | 0.003 | -0.0003 | 3.85 | 0.023 | 0.00099 | 3.20 | 0.0006 | 0.000059* | 3.17 |
|  | Seed length | 0.036 | 0.00043* | 3.19 | 0.014 | 0.00073* | 3.39 | 0.0025 | -0.00069 | 4.56 | 0.000068 | -0.00032 | 3.65 | 0.0028 | -0.00011 | 3.92 |
| TCM | Leaf length | 0.025 | 0.13* | 9.74 | 0.00011 | -0.0043* | 10.97 | 0.0059 | -0.039 | 10.55 | 0.023 | -0.085 | 12.14 | 0.14* | 0.21* | 18.71 |
|  | Leaf width | 0.0047 | 0.0032 | 0.83 | 0.031* | -0.0060 | 0.86 | 0.00024 | 0.0019 | 0.90 | 0.002 | 0.0016 | 0.64 | 0.29* | -0.021* | 0.37 |
|  | Seed cone length | 0.19* | 2.21 | 60.72 | 0.17* | 1.69 | 86.54 | 0.0011 | 0.15* | 66.29 | 0.27* | 1.85 | 81.17 | 0.024 | 0.28* | 43.45 |
|  | Seed cone diameter | 0.30* | 1.29* | 37.87 | 0.098* | 0.46 | 43.28 | 0.0015 | -0.019* | 36.83 | 0.27* | 0.55 | 35.76 | 0.04 | 0.16* | 25.61 |
|  | Seed scale length | 0.04 | 0.17 | 13.08 | 0.004 | 0.037 | 15.21 | 0.006 | -0.063 | 14.02 | 0.02 | 0.046 | 14.92 | 0.062 | -0.083* | 9.31 |
|  | Seed scale width | 0.002 | -0.033 | 8.82 | 0.01 | 0.048 | 11.35 | 0.002 | -0.033 | 10.59 | 0.06 | 0.13 | 10.78 | 0.033 | -0.0066* | 6.47 |
|  | Seed wing length | 0.00054 | 0.011 | 7.48 | 0.13* | 0.18 | 9.97 | 0.012 | -0.056 | 8.11 | 0.032 | 0.074 | 11.77 | 0.042 | -0.022 | 8.04 |
|  | Seed wing width | 0.037 | -0.045 | 2.96 | 0.04* | 0.034* | 3.97 | 0.0085 | -0.019 | 3.52 | 0.0047 | 0.013 | 4.21 | 0.0006 | -0.0016* | 3.15 |
|  | Seed length | 0.045 | -0.058 | 3.12 | 0.031* | 0.024 | 4.05 | 0.0059 | -0.013 | 3.91 | 0.0088 | -0.0011 | 3.62 | 0.0002 | 0.00069 | 2.86 |
| TWM | Leaf length | 0.0015 | 0.21 | 86.06 | 0.0022 | -0.021** | 109.95 | 0.024 | 0.63* | 100.87 | 0.0014 | 0.018 | 121.88 | 0.21** | 9.79* | -55.79 |
|  | Leaf width | 0.0014 | -0.0016 | 0.81 | 0.00073 | 0.00094 | 0.86 | 0.032 | -0.00058 | 0.95 | 0.039 | 0.0057 | 0.57 | 0.021 | -0.025 | 1.19 |
|  | Seed cone length | 0.086* | 1.05* | 34.93 | 0.029* | 0.925** | 73.26 | 0.012 | 0.39* | 61.43 | 0.0016 | -0.12 | 82.8 | 0.0034 | 0.46 | 28.11 |
|  | Seed cone diameter | 0.0083 | 0.15 | 26.77 | 0.0015 | 0.073 | 41.98 | 0.025 | 0.21* | 34.76 | 0.021 | 0.12* | 34.29 | 0.064 | 0.92 | 3.37 |
|  | Seed scale length | 0.0035 | -0.032 | 12.08 | 0.021 | -0.11 | 16.50 | 0.065* | 0.17* | 19.74 | 0.035 | 0.05** | 12.13 | 0.077 | -0.23** | 13.81 |
|  | Seed scale width | 0.0048 | 0.033 | 8.76 | 0.0056 | -0.042 | 11.93 | 0.021 | 0.083 | 9.91 | 0.026 | 0.07 | 9.55 | 0.013 | -0.061 | 7.85 |
|  | Seed wing length | 0.0015 | 0.014 | 7.33 | 0.032 | 0.11 | 8.48 | 0.028 | 0.071 | 7.67 | 0.054 | -0.074 | 12.91 | 0.0036 | -0.0094 | 8.64 |
|  | Seed wing width | 0.036 | 0.0259 | 3.03 | 0.021 | 0.029 | 3.55 | .035 | 0.32 | 3.29 | 0.0012 | 0.0016 | 4.15 | 0.012 | -0.032 | 3.83 |
|  | Seed length | 0.00149 | -0.027 | 3.82 | 0.013 | 0.022 | 3.72 | 0.010 | 0.015 | 3.83 | 0.036 | -0.017 | 3.96 | 0.0019 | -0.010 | 3.07 |
| α | Leaf length | 0.0112 | 1.78 | 8.16 | 0.00083 | 0.44 | 10.76 | 0.00026 | -0.0083 | 10.77 | 0.0064 | -1.26 | 12.86 | 0.18* | 29.99* | 6.21 |
|  | Leaf width | 0.0024 | 0.046* | 0.79 | 0.009 | -0.11* | 0.91 | 0.0069 | -0.034 | 0.91 | 0.0047 | -0.022 | 0.65 | 0.07 | -1.30 | 1.65 |
|  | Seed cone length | 0.015 | 12.17 | 39.85 | 0.083* | 45.09* | 63.34 | 0.0089 | 13.97 | 60.13 | 0.27* | 51.97* | 52.63 | 0.022 | 34.20 | 14.34 |
|  | Seed cone diameter | 0.0021 | -2.17 | 28.73 | 0.063* | 12.91* | 36.68 | 0.0015 | 2.08 | 36.13 | 0.25* | 15.02* | 27.51 | 0.11* | 33.48* | 1.02 |
|  | Seed scale length | 0.0017 | -0.65 | 11.99 | 0.0035 | -0.041 | 15.2 | 0.0039 | -1.48 | 14.92 | 0.022 | 0.14 | 12.2 | 0.016 | 5.52 | 6.88 |
|  | Seed scale width | 0.034 | 2.66* | 8.18 | 0.016 | 0.69* | 10.97 | 0.0058 | -0.55 | 10.98 | 0.039 | 2.93 | 9.15 | 0.012 | -1.61 | 7.73 |
|  | Seed wing length | 0.011 | -1.02 | 7.72 | 0.089* | 5.19* | 7.33 | 0.0063 | -1.36 | 8.92 | 0.019 | 1.53 | 10.91 | 0.0013 | -0.49 | 8.78 |
|  | Seed wing width | 0.019 | 0.66 | 3.08 | 0.02 | 0.95* | 3.49 | 0.0027 | -0.36 | 3.75 | 0.0051 | 0.038 | 4.18 | 0.0041 | -0.51 | 3.53 |
|  | Seed length | 0.13* | -1.93 | 4.19 | 0.016 | 0.78* | 3.65 | 0.0064 | 0.048 | 3.96 | 0.0045 | -0.022 | 3.63 | 0.003 | -0.39 | 3.13 |

**Figure S1**. The reconstruction phylogeny tree of 10 spruce species.


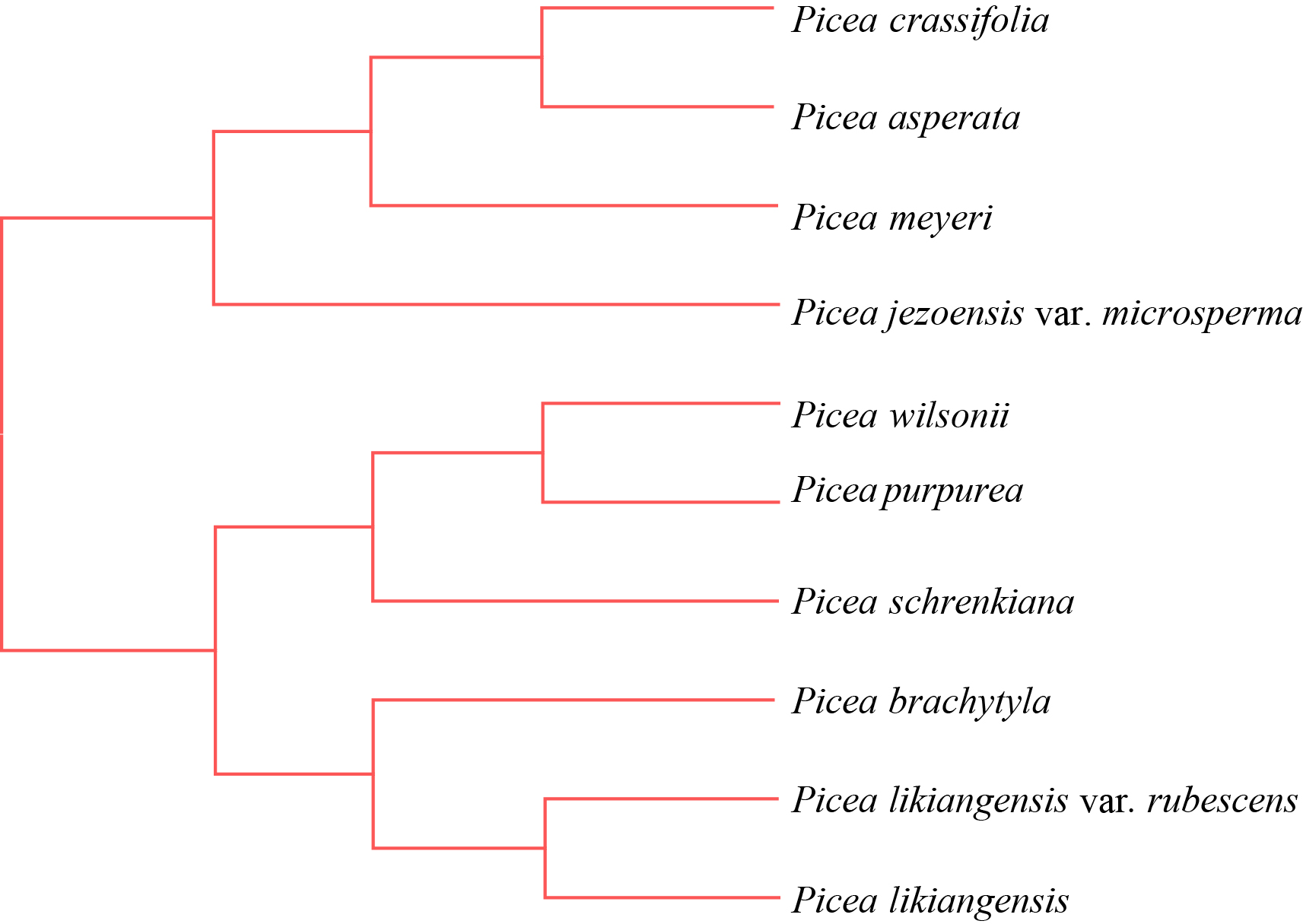


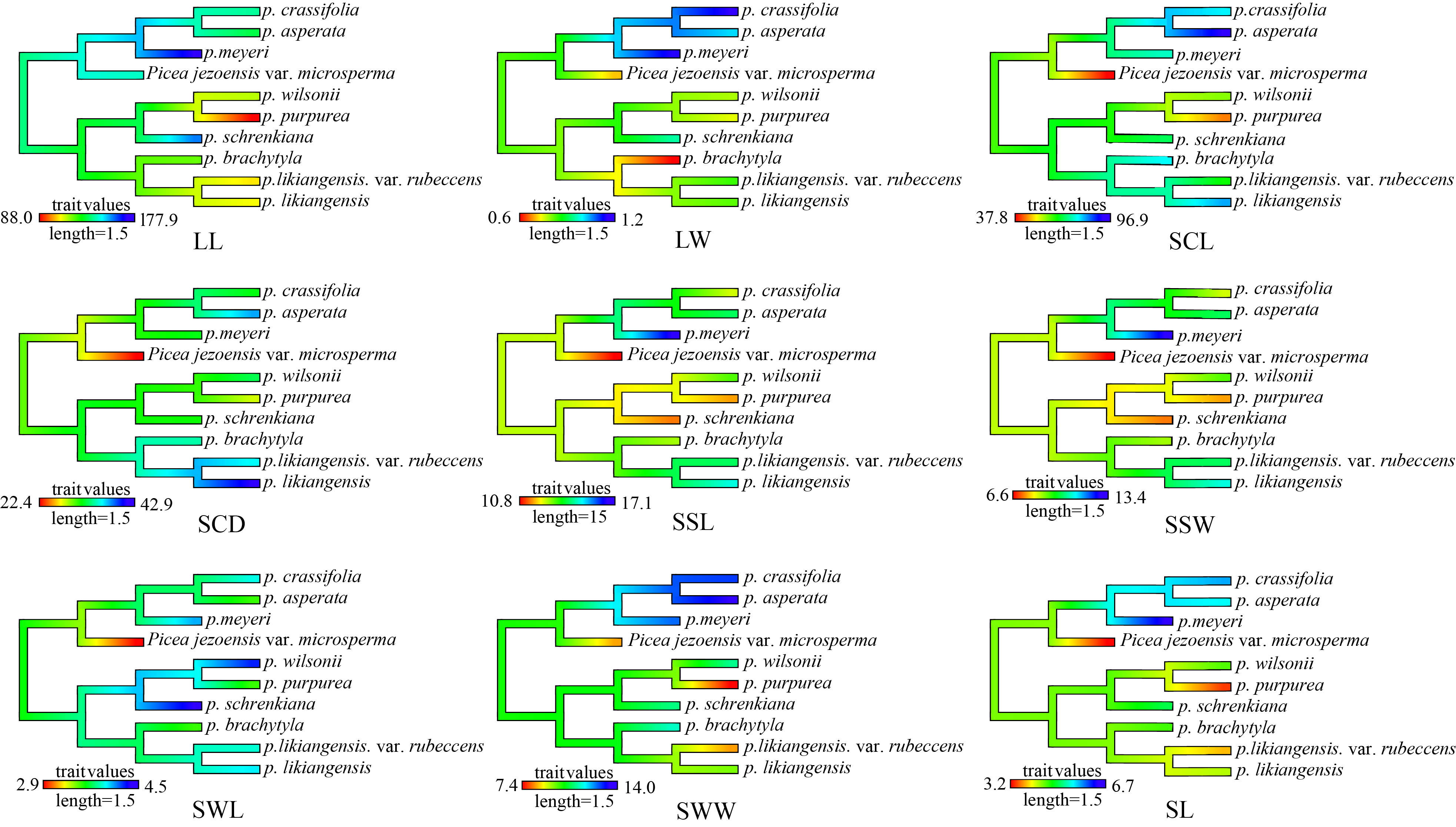
**Figure S2.** ContMap of the mean values of spruce traits at each order. LL, Leaf length (mm); LW, Leaf width (mm); SCL, Seed cone length (mm); SCD, Seed cone diameter (mm); SSL, Seed scale length (mm); SCW, Seed scale width (mm); SWL, Seed wing length (mm); SWW, Seed wing width (mm); and SL, Seed length (mm).
